# Cost-effectiveness of response evaluation after chemoradiation in patients with advanced oropharyngeal cancer using ^18^F–FDG-PET-CT and/or diffusion-weighted MRI

**DOI:** 10.1186/s12885-017-3254-0

**Published:** 2017-04-11

**Authors:** Marjolein JE Greuter, Charlotte S Schouten, Jonas A Castelijns, Pim de Graaf, Emile FI Comans, Otto S Hoekstra, Remco de Bree, Veerle MH Coupé

**Affiliations:** 1 Department of Epidemiology and Biostatistics, VU University Medical Center, PO Box 7057, MF F-wing ST, 1007 MB Amsterdam, the Netherlands; 2 Department of Otolaryngology-Head and Neck Surgery, VU University Medical Center, PO Box 7057, 1007 MB Amsterdam, the Netherlands; 3 Department of Otorhinolaryngology-Head and Neck Surgery, Radboud University Medical Center Nijmegen, PO Box 9101, 6500 HB Nijmegen, the Netherlands; 4 Department of Radiology and Nuclear medicine, VU University Medical Center, PO Box 7057, 1007 MB Amsterdam, the Netherlands; 5 Department of Head and Neck Surgical Oncology, UMC Utrecht Cancer Center, University Medical Cancer Utrecht, PO Box 85500, 3508 GA Utrecht, the Netherlands

**Keywords:** Oropharyngeal cancer, Response evaluation, Cost-effectiveness, ^18^F-FDG-PET-CT, Diffusion-weighted MRI

## Abstract

**Background:**

Considerable variation exists in diagnostic tests used for local response evaluation after chemoradiation in patients with advanced oropharyngeal cancer. The yield of invasive examination under general anesthesia (EUA) with biopsies in all patients is low and it may induce substantial morbidity. We explored four response evaluation strategies to detect local residual disease in terms of diagnostic accuracy and cost-effectiveness.

**Methods:**

We built a decision-analytic model using trial data of forty-six patients and scientific literature. We estimated for four strategies the proportion of correct diagnoses, costs concerning diagnostic instruments and the proportion of unnecessary EUA indications. Besides a reference strategy, i.e. EUA for all patients, we considered three imaging strategies consisting of ^18^FDG-PET-CT, diffusion-weighted MRI (DW-MRI), or both ^18^FDG-PET-CT and DW-MRI followed by EUA after a positive test. The impact of uncertainty was assessed in sensitivity analyses.

**Results:**

The EUA strategy led to 96% correct diagnoses. Expected costs were €468 per patient whereas 89% of EUA indications were unnecessary. The DW-MRI strategy was the least costly strategy, but also led to the lowest proportion of correct diagnoses, i.e. 93%. The PET-CT strategy and combined imaging strategy were dominated by the EUA strategy due to respectively a smaller or equal proportion of correct diagnoses, at higher costs. However, the combination of PET-CT and DW-MRI had the highest sensitivity. All imaging strategies considerably reduced (unnecessary) EUA indications and its associated burden compared to the EUA strategy.

**Conclusions:**

Because the combined PET-CT and DW-MRI strategy costs only an additional €927 per patient, it is preferred over immediate EUA since it reaches the same diagnostic accuracy in detecting local residual disease while leading to substantially less unnecessary EUA indications. However, if healthcare resources are limited, DW-MRI is the strategy of choice because of lower costs while still providing a large reduction in unnecessary EUA indications.

## Background

Patients with resectable advanced staged oropharyngeal squamous cell carcinoma (OPSCC) are often treated with chemoradiation (CRT) in order to preserve organ function and quality of life. Low residual and recurrent tumour rates indicate that CRT is an adequate treatment option [1]. Still, thorough follow-up is warranted to detect residual tumour, which can be successfully treated with salvage surgery if detected early. Previous research has shown that early detection of residual tumour is associated with more favourable survival probabilities and better local control [1, 2]. Thus, timely detection of residual tumour is essential.

A Dutch survey showed that there is considerable variation in response evaluation after CRT, especially in the diagnostic tests performed [3]. Tests that are used are examination under general anesthesia with taking of biopsies (EUA), computed tomography (CT), magnetic resonance imaging (MRI) and ^18^F–fluorodeoxy-glucose positron emission tomography combined with CT (^18^F–FDG-PET-CT). EUA is considered to be the most reliable procedure to detect residual disease but is an invasive procedure during which biopsies are taken in areas treated with CRT. Besides the risk of side-effects such as pain, inflammation and wound healing problems, there is also the possibility of sampling error leading to false-negative results [1]. Furthermore, EUA has considerable impact on scarce resources because hospital stay and operating facilities are required.

In contrast to EUA, imaging tests are not invasive. However, conventional CT and MRI may not be suitable because postradiation effects, e.g. fibrosis and necrosis [4], may hamper accurate interpretation of the images. More advanced tests such as ^18^F–FDG-PET-CT and diffusion-weighted MRI (DW-MRI) may be more suitable options. These tests do not only assess if any anatomical residual mass is present, but also determine the metabolic activity and cell density, respectively, of the tumour.

PET-CT and DW-MRI cannot completely replace EUA because pathological confirmation is required before further treatment. Nevertheless, they could be used to select those patients with a high risk of residual disease for further diagnostic workup with EUA. This would reduce the number of patients that have to undergo futile invasive diagnostic procedures. Furthermore, the imaging results provide the opportunity to guide biopsy procedures, thereby possibly reducing sampling error. Besides these advantages for patients, imaging to select patients for EUA might also lead to cost reductions. Therefore, this study aimed to assess the effects and costs of four response evaluation strategies to detect local residual disease, namely EUA for all patients, PET-CT-based selection for EUA, DW-MRI-based selection for EUA and a combination of PET-CT and DW-MRI to select for EUA. All analyses were conducted using a decision-analytic model based on trial data of forty-six patients and scientific literature.

## Methods

### Strategies

We developed a decision-analytic model to evaluate the effects and costs of four strategies for local response evaluation in patients with OPSCC who underwent CRT. In the first strategy under consideration, i.e. the reference strategy, all patients undergo EUA 3 months after completion of CRT for response evaluation. EUA is considered positive if histopathological proof of viable cancer has been found in the biopsy. The indication for EUA is considered unnecessary if 6 months of follow-up after response evaluation did not show residual disease.

**Fig. 1:**
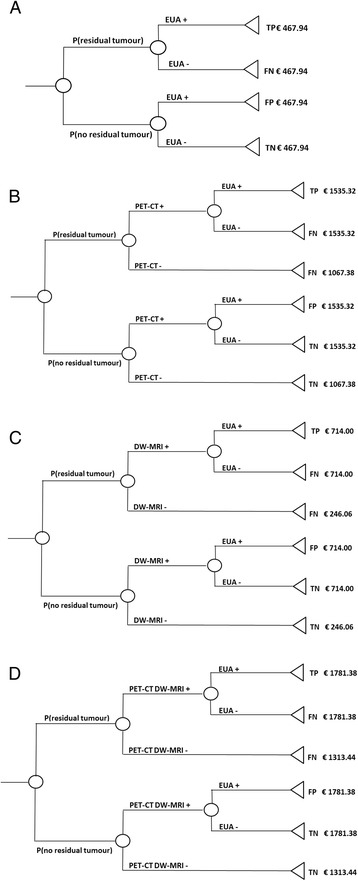
Decision trees of all evaluated strategies namely (**a**) EUA for all patients, (**b**) PET-CT prior to EUA, (**c**) DW-MRI prior to EUA and (**d**) PET-CT as well as DW-MRI prior to EUA. The end node depicts the diagnostic status of the patient, i.e. true positive (TP), false negative (FN), false positive (FP) or true negative (TN), as well as the costs associated with that specific branch

In the three other strategies, response evaluation is performed by either PET-CT (strategy 2), DW-MRI (strategy 3) or a combination of PET-CT and DW-MRI (strategy 4). Only patients with a positive imaging test are referred to EUA. In strategy 4, patients are referred to EUA based on a combined assessment by a radiologist and nuclear medicine physician. The four strategies are depicted by decision trees in Fig. 1. Each end node reflects the diagnostic status of a patient who followed that specific branch as well as the associated costs.

### Data

Costs of the different diagnostic instruments were based on official tariff lists [5]. As shown in Table 1, combined PET-CT and DW-MRI was with 1313 Euros the most expensive test. Test characteristics were obtained from a prospective trial (2012–2014) in which patients with advanced but resectable OPSCC who underwent CRT were subjected to PET-CT, DW-MRI and EUA for response evaluation 3 months after end of CRT (Dutch Trial Register: NTR4111/NL39181.000.11). Patient and tumour characteristics are summarized in Table 2. PET-CT and DW-MRI images were interpreted by respectively two nuclear medicine physicians (OH, EC) and two radiologists (JC, PG). For PET-CT, we requested the observers individually to score the PET-CT using the Hopkins criteria (a 5-point scale in which scores 1, 2 and 3 are considered negative for tumour and scores 4 and 5 are considered positive) [6]. DW-MRI observers scored signal intensity on b1000 images and the corresponding ADC-map. A residual mass was considered ‘suspicious for residual disease’ if increased signal intensity on the b1000 images was present, in an area of contrast-enhancement on post-contrast T1WI and corresponding to low ADC-values. For DW-MRI, we requested the observers individually to classify the MRI-examinations using a dichotomous system, i.e. suspicious for a local residue or not, based on signal intensity of the DW-images. In case readings were discrepant, consensus was reached between the observers of either PET-CT or DW-MRI. For strategy 4, combined reading of PET-CT and DW-MRI with visual correlation by a radiologist and nuclear medicine physician was performed in all patients with discrepancies between the scoring of the two modalities. In total, 46 patients were included of whom five (11%) had residual tumour. Test characteristics of each diagnostic test are shown in Table 2. Sensitivities were 60% or higher, with the combined PET-CT and DW-MRI strategy having the highest sensitivity. Specificity was lowest for PET-CT with 83%.

**Table 1:** Test characteristics and costs of the diagnostic instruments for response evaluation. In the base-case analysis, trial-reported test characteristics were used

	Trial-reported test characteristics				Test characteristic based on literature		Costs (Euros)
	Sensitivity [95% CI]	Specificity [95% CI]	Positive predictive value [95% CI]	Negative predictive value [95% CI]	Sensitivity	Specificity	
EUA	60 [14.7–94.7]	100 [91.4–100.0]	100.0 [29.2–100.0]	95.3 [84.2–99.4]	*	*	468
PET-CT	75 [19.4–99.4]	83 [67.9–92.8]	30.0 [6.7–65.2]	97.1 [85.1–99.9]	79.9^a^	87.5^a^	1067
DW-MRI	60 [14.7–94.7]	95 [83.5–99.4]	60.0 [14.7–94.7]	95.1 [83.5–99.4]	89.0^b^	86.0^b^	246
Combined PET-CT and DW-MRI	100 [39.8–100.0]	93 [80.1–98.5]	57.1 [18.4–90.1]	100.0 [90.7–100.0]	*	*	1313

^*^Test characteristics for this diagnostic instrument are not reported in the literature

^a^Based on the systematic review of Gupta et al. (2011) [7]

^b^Based on the study of Vaid et al. (2017) [8]

**Table 2:** Patient and tumour characteristics

Characteristic	No. of patients (%) (*n* = 46)
Gender	
Male	35 (76.1%)
Female	11 (23.9%)
Mean age at diagnosis, years (range)	60.4 (44–71)
Oropharyngeal subsite	
Base of tongue	22 (47.8%)
Tonsil	19 (41.3%)
Oropharynx nos	5 (10.9%)
HPV-status	
Positive	20 (43.5%)
Negative	26 (56.5%)
T-stage	
1–2	20 (43.5%)
3	12 (26.1%)
4a	14 (30.4)
N-stage	
0	5 (10.9%)
1	10 (21.7%)
2a	2 (4.3%)
2b	20 (43.5%)
2c	9 (19.6%)
M-stage	
0	46 (100%)
1	0 (0%)
Smoking	
Never (0–5 pack years)	9 (19.6%)
Moderate (6–24 pack years)	8 (17.4%)
Heavy (>24 pack years)	29 (63.0%)
Alcohol consumption	
Never (0)	3 (6.5%)
Moderate (1–149 unit years)	25 (54.3%)
Heavy (>149 unit years)	18 (39.1%)

Smoking was defined in pack years (1 pack year = 20 cigarettes a day during 1 year)

Alcohol consumption was defined in unit years (1 unit year = one alcohol-containing consumption a day during 1 year)

*Abbreviations*: *CRT* chemoradiotherapy, *HPV* Human papillomavirus, *no* number, *nos* not otherwise specified

### Analyses

Using the decision trees, we calculated the expected health effects of each response evaluation strategy, i.e. the expected proportion of correctly diagnosed patients (true positives and true negatives). Based on the costs of each diagnostic instrument, the average costs accumulated by a patient when following a specific branch in the decision tree were estimated. Subsequently, for each branch costs were multiplied by the probability that a patient will follow this branch. Summing up the expected costs of each branch of the decision tree results in the overall expected costs for that strategy. In addition, the number of EUA indications as well as the number of unnecessary EUA indications per response evaluation strategy were calculated. It was not possible to correctly estimate quality-adjusted life-years in this study. As an alternative we estimated the costs per true-positive case for the different strategies.

### Sensitivity analyses

Due to the small sample size of this trial, we repeated the base-case analysis using test characteristics based on the literature. Sensitivity and specificity for PET-CT and DW-MRI were derived from respectively a systematic review by Gupta et al. (2011) [7] and the study of Vaid et al. (2017) [8]. Test characteristics for EUA and combined PET-CT and DW-MRI are not reported in the literature.

We further explored the impact of uncertainty regarding the test characteristics of the diagnostic instruments in univariate sensitivity analyses in which we increased and decreased the sensitivity and specificity of each instrument with 10% (absolute change). In the strategies in which a positive imaging test was followed by EUA, only the test characteristics of the imaging test were varied.

In addition, in clinical practice a positive EUA is usually followed by MRI and PET-CT to assess if surgical intervention is feasible and if any distant metastases are present, respectively. Therefore, in a sensitivity analysis we included the costs of these additional tests in the EUA strategy. Similarly, we added costs of an MRI or whole body PET-CT (in case of a positive EUA) to those of the “head and neck” PET-CT and DW-MRI, respectively. Finally, we assessed the impact of uncertainty surrounding the prevalence of residual tumour by increasing and decreasing the prevalence to 15% and 5%, respectively [9].

## Results

### Base-case analysis

The proportion of correctly diagnosed patients and the expected costs per patient for each strategy are shown in Table 3. The combined PET-CT and DW-MRI strategy and the EUA strategy led to the highest proportion of correctly diagnosed patients, i.e. 96%. However, the expected costs of the EUA strategy were 927 Euros per patient lower than those of the combined PET-CT and DW-MRI strategy. The strategy with DW-MRI led to the lowest expected costs per patient but also to the lowest proportion of correct diagnoses. The PET-CT strategy and the combined PET-CT and DW-MRI strategy were dominated by the EUA strategy because these strategies led to a smaller or equal proportion of correct diagnoses, at higher costs.

**Table 3:** Expected proportion of correctly classified patients, expected costs per patient and proportion of unnecessary EUA indications per strategy

Strategy	Expected proportion of correctly classified patients	Expected costs	Costs per true-positive case	Proportion of unnecessary EUA indications
Base-case analysis				
*EUA*	0.96	468	7175	0.89
*PET-CT*	0.94	1177	24,058	0.65
*DW-MRI*	0.93	297	7588	0.40
*Combined PET-CT and DW-MRI*	0.96	1395	21,387	0.38
Test characteristics based on the literature				
*PET-CT*	0.94	1160	22,264	0.56
*DW-MRI*	0.95	350	6025	0.56
Sensitivity +10%				
*EUA*	0.97	468	6150	0.89
*PET-CT*	0.96	1207	18,352	0.60
*DW-MRI*	0.94	302	6615	0.36
*Combined PET-CT and DW-MRI* ^a^	0.96	1395	21,387	0.38
Specificity +10%				
*EUA*	0.96	468	7175	0.89
*PET-CT*	0.94	1135	23,205	0.44
*DW-MRI*	0.93	277	7068	0
*Combined PET-CT and DW-MRI*	0.96	1364	20,919	0
Sensitivity −10%				
*EUA*	0.95	468	8610	0.89
*PET-CT*	0.93	1172	27,639	0.68
*DW-MRI*	0.92	292	8950	0.44
*Combined PET-CT and DW-MRI*	0.95	1390	23,677	0.40
Specificity −10%				
*EUA*	0.87	468	7175	0.89
*PET-CT*	0.94	1218	24,910	0.75
*DW-MRI*	0.93	339	8654	0.67
*Combined PET-CT and DW-MRI*	0.96	1437	22,027	0.59
EUA followed by additional tests				
*EUA*	0.96	574	8799	0.89
*PET-CT*	0.94	1189	24,304	0.65
*DW-MRI*	0.93	351	8966	0.40
Increasing the prevalence of residual disease				
*EUA*	0.94	9834	5199	0.85
*PET-CT*	0.92	10,554	17,599	0.56
*DW-MRI*	0.90	9673	5696	0.32
*Combined PET-CT and DW-MRI*	0.94	10,778	15,697	0.29
Decreasing the prevalence of residual disease				
*EUA*	0.98	7290	15,598	0.95
*PET-CT*	0.97	7982	51,592	0.81
*DW-MRI*	0.97	7103	15,655	0.61
*Combined PET-CT and DW-MRI*	0.98	8191	45,645	0.58

^a^Sensitivity was 100% in base-case analysis

### Number of EUA indications and missed residual disease

**Fig. 2:**
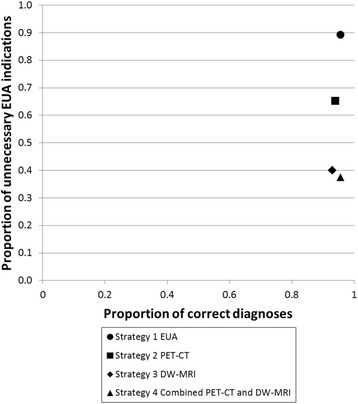
Proportion of correct diagnoses and unnecessary EUA indications per strategy

Figure 2 shows the proportion of correct diagnoses as well as the proportion of unnecessary EUA indications per strategy. All imaging strategies led to a considerably lower proportion of unnecessary EUA indications compared to the EUA strategy. The combined PET-CT and DW-MRI strategy led to the lowest proportion of unnecessary EUA indications, i.e. 38%.

**Fig. 3:**
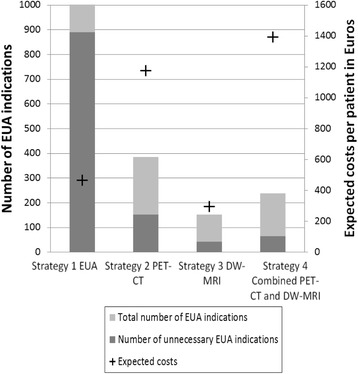
Total number of EUA indications, number of unnecessary EUA indications and expected costs per patient per strategy for 1000 patients

In Fig. 3, the total number of EUA indications, the number of unnecessary EUA indications and the expected costs per patient are shown for 1000 patients. In the EUA strategy, all patients underwent EUA, of which 891 indications were unnecessary. All imaging strategies substantially reduced the number of required EUAs as well as the number of unnecessary EUA indications. The DW-MRI required the lowest number of EUAs, i.e. 109 EUAs of which 43 indications were unnecessary. However, this strategy also had the highest number of missed residual disease. Please note that the combined PET-CT and DW-MRI required 65 additional EUAs compared to the DW-MRI strategy, but a slightly lower proportion of these EUA indications was unnecessary.

The costs per true-positive case were for EUA 7175 Euros, for PET-CT 24,058 Euros, for DW-MRI 7588 Euros and for combined PET-CT and DW-MRI 21,387 Euros. Although EUA and DW-MRI are much cheaper, they detect less recurrences than PET-CT leading to delayed salvage treatment with potentially poorer outcome. The addition of DW-MRI to PET-CT lowered the costs per true-positive case.

### Univariate sensitivity analyses

When the test characteristics of DW-MRI were based on the literature, the proportion of correct diagnoses increased from 0.93 to 0.95. On the other hand, also the expected costs and proportion of unnecessary EUA indications increased. For the PET-CT strategy, the proportion of correct diagnoses remained 0.95 whereas the expected costs and proportion of unnecessary EUA indications decreased when we based the test characteristics on the literature.

In the majority of sensitivity analyses in which we varied test characteristics, additional testing after a positive EUA and the prevalence of residual disease, the ordering of the strategies based on the proportion of correct diagnoses, expected costs and proportion of unnecessary EUA indications did not change, as shown in Table 3. Only in the analysis in which the specificity of the diagnostic instruments was lowered with 10%, the ordering of the strategies changed; the EUA strategy led to the lowest proportion of correct diagnoses.

## Discussion

This study explored the cost-effectiveness of four strategies used for response evaluation to detect local residual disease after CRT in patients with advanced staged OPSCC. In the EUA strategy, i.e. the reference strategy, 96% of patients were correctly diagnosed. Expected costs were 468 Euros at the expense of 89% unnecessary EUA indications. The DW-MRI strategy was with 297 Euros the least costly strategy. However, this strategy also led to the lowest proportion of correct diagnoses, i.e. 93%. The PET-CT strategy and the combined PET-CT and DW-MRI strategy were dominated by the EUA strategy due to a smaller or equal proportion of correct diagnoses, at higher costs. All imaging strategies considerably reduced the number of EUA indications and unnecessary EUA indications compared to the EUA strategy.

Based on our model results, the combined PET-CT and DW-MRI strategy is preferred over the EUA strategy. This strategy has the same diagnostic accuracy as immediate EUA while considerably reducing the number of EUA indications as well as unnecessary EUA indications. On the other hand, the combined imaging strategy costs an additional 927 Euros. However, only around 220 patients are diagnosed each year with advanced OPSCC in the Netherlands [10]. Thus, these additional costs are negligible on a society level. Furthermore, if all 220 patients were evaluated based on the combined imaging strategy, only 38 EUAs are indicated of which 14 would be unnecessary. When the EUA strategy would be used for response evaluation, 220 EUAs are indicated of which 196 would be unnecessary.

When healthcare resources are limited, our study suggests that DW-MRI prior to EUA is the strategy of choice. This strategy is less costly than both the combined PET-CT and DW-MRI strategy and EUA strategy. Furthermore, it has a lower impact on scarce resources such as hospital stay compared to immediate EUA. Moreover, fewer patients are exposed to invasive EUA and of the EUA indications, a lower proportion is unnecessary. However, the proportion of correct diagnoses is lower than in the EUA strategy. This difference is caused by a higher proportion of false-negative test results meaning that residual tumour is missed.

Besides decreasing the number of unnecessary EUA indications, another possible advantage of imaging is that the results of the imaging test can be used to guide the biopsy taking. This may increase the sensitivity of EUA. As the surgeons in the trial were not blinded to the imaging results, it may be possible that the sensitivity of EUA without prior imaging is overestimated. We assessed the impact of this in sensitivity analyses by assuming a 10% lower sensitivity in the EUA strategy. This changed the ordering of the strategies; the combined PET-CT and DW-MRI strategy became the strategy with most correct diagnoses. Nevertheless, EUA detected only 60% of the residual tumours, questioning the degree of bias.

The test characteristics of PET-CT and DW-MRI were based on a trial in which all patients received PET-CT, DW-MRI and EUA 3 months after CRT. Timing is an important determinant of these test characteristics. If the test is conducted too soon after treatment, post-radiation effects can lead to a false-positive test. Also false-negative test results are possible because tumour cells have not yet reached a detectable size. On the other hand, early diagnosis of residual disease increases treatment success of salvage surgery [11]. A study indicated that DW-MRI may be used for response evaluation 3 weeks after CRT [12]. The optimal timing of imaging has still to be determined, which might influence test characteristics.

Furthermore, DW-MRI is a relative new imaging technique within oncological applications and therefore, little experience is gained with DW-MRI in the post-treatment evaluation so far. Besides, DW-imaging is susceptible to artefacts, particularly in the inhomogeneous head and neck area which contains a variety of tissues. Also, geometrical distortions due to interfaces between soft tissue and air or bone can occur. Although DW-MRI is not yet an established technique for response evaluation, this early assessment of the expected health effects and costs provides more insight in the potential of DW-MRI as a response evaluation strategy. We showed that DW-MRI is the strategy of choice when combined with PET-CT. However, a response evaluation strategy solely based on DW-MRI followed by EUA in individuals with a positive imaging test is only preferred in settings with limited health care resources. Nevertheless, radiologists are still in the learning curve concerning post-treatment evaluation of DW-MRI. With more training and feedback, DW-MRI is expected to obtain a higher accuracy. The impact of increased sensitivity was assessed in sensitivity analyses, but conclusions did not change.

Another emerging technique for response evaluation is PET-MRI. This technique combines the often complementary data from PET and MRI and could lead to improved anatomic localisation of focal uptake compared to PET-CT [13]. This could potentially decrease the number of false-positive test outcomes and as a consequence, reduce the number of unnecessary EUA indications.

The trial included only 46 patients of whom five were diagnosed with residual disease. Due to this small sample size, there was a fair amount of uncertainty regarding test characteristics and the prevalence of residual disease. Repeating the base-case analysis using test characteristics derived from the literature did not change our conclusion. Furthermore, our residual primary tumour rate of 11% was in agreement with the results of Moeller et al. (2009) [14]. On the other hand, Van den Broek et al. (2006) reported a residual primary tumour rate of 7% [1]. We have addressed this issue by varying the prevalence of residual disease in one-way sensitivity analyses. However, the ordering of the strategies based on correct diagnoses, costs and unnecessary EUA indications did not change.

In this cohort of patients, the residual primary tumour rate was only 11%. To improve the yield of routine response evaluation, only patients with high risk factors should undergo this diagnostic procedure. Risk factors which can be used to select patients include T-stage [15], HPV (human papilloma virus) status [16, 17] and pre-treatment metabolic tumour volume [18].

We did not differentiate between HPV-related and HPV-unrelated tumours. For HPV-related tumours, a lower residual disease rate is observed which is probably due to higher responsiveness to chemoradiation [19, 20]. However, it is unclear whether HPV-related tumours have the same probability of being detected as HPV-unrelated tumours. In this study, 44% of the tumours were HPV positive whereas all the patients with residual disease had a HPV negative tumour. Nevertheless, the small sample size precludes definite conclusions regarding differences in detection.

Outcomes of this study were the proportion of correctly diagnosed patients, costs concerning diagnostic instruments and the number of unnecessary EUA indications. This means that health benefits, i.e. the proportion of correct diagnoses, and treatment burden, i.e. unnecessary EUA indications, were evaluated separately. Moreover, not all health benefits and treatment burden were captured in these outcomes. For example, declined survival probabilities due to false-negative test results as well as side-effects of EUA were not taken into account. Outcomes that encompass all health benefits and treatment burden such as quality-adjusted life-years (QALYs) would therefore be preferable. Comparing costs per QALY would lead to a more comprehensive evaluation of the response evaluation strategies. However, it was not possible to calculate QALYs due to the trial design. Patients in the trial were subjected to all tests for response evaluation meaning that residual disease which may be missed by one test (false-negative) could be detected by one of the other tests. If patients would be subjected to only one test, as in the strategies evaluated in this study, patients with false-negative test results would have become symptomatically detected at a later point of time. Since earlier detection leads to improved survival probabilities [1, 2], it was not possible to correctly estimate QALYs. As an alternative, we calculated costs per true-positive case for the different strategies.

We hypothesize that the ordering of strategies would not change when the evaluation was based on costs per QALY. A strategy using an instrument with a high sensitivity and few (unnecessary) EUA indications would be favoured because this strategy would lead to low rates of missed residual disease and low treatment burden. This means that the combined PET-CT and DW-MRI strategy would still be the strategy of choice. To assess this hypothesis, future studies should include costs per QALY.

To our knowledge, few studies have assessed the cost-effectiveness of response evaluation in patients with head and neck cancer treated with CRT. Two studies compared a strategy of PET-CT scanning prior to neck dissection and up-front neck dissection for all patients. These studies showed that the imaging strategy was more cost-effective [9, 21]. Although these studies did not compare imaging to EUA, they also indicate that imaging can be more cost-effective than more invasive strategies.

In clinical practice, PET-CT can be used for response evaluation at the primary site and neck and to detect distant metastases simultaneously. Previous studies have shown that PET-CT is cost-effective for response evaluation after (chemo)radiation of the pretreatment advanced stage positive neck when only patients with a PET-CT positive neck underwent neck dissection compared to planned neck dissection in all patients [21, 22]. Moreover, pretreatment screening for distant metastases using PET-CT appeared also to be cost-effective [23]. These evaluations by PET-CT add to the cost-effectiveness of response evaluation of the primary site as reported in the present study. For DW-MRI no data on cost-effectiveness in response evaluation of neck disease is available. Also for response evaluation of advanced nodal neck disease the combination of PET-CT and DW-MRI seems to have the highest sensitivity and specificity [24].

Results of cost-effectiveness studies can be used for the development of a guideline for response evaluation in patients with OPSCC. The need for such a guideline is underlined by a previous study showing that there is substantial variation in the diagnostic tests used for response evaluation [3]. By including studies on cost-effectiveness in the guideline development process, guideline recommendations will not only be based on the most effective strategy, but on the most cost-effective strategy. This will lead to more sensible use of scarce healthcare resources. This is the first cost-effectiveness study evaluating different response evaluation strategies. However, there was considerable uncertainty regarding important model parameters due to the small sample size of the trial. Additional studies, preferably based on trials with a larger sample size, are required to provide a comprehensive evidence base for guideline development.

## Conclusions

This study suggests that combined PET-CT and DW-MRI is the strategy of choice for local response evaluation. It is preferred over immediate EUA since it costs only an additional €927 while reaching the same diagnostic accuracy and leading to substantially less unnecessary EUA indications and its associated burden. However, if healthcare resources are limited, DW-MRI is the preferred strategy because of lower costs while maintaining the number of unnecessary EUA indications low.

## Abbreviations

18F–FDG-PET-CT: 18F–fluordeoxy-glucose positron emission tomography combined with computed tomography
CRT: chemoradiation
CT: computed tomography
DW-MRI: diffusion-weighted magnetic resonance imaging
EUA: examination under general anesthesia
MRI: magnetic resonance imaging
OPSCC: oropharyngeal squamous cell carcinoma
QALY: quality-adjusted life-years
